# IL-10 Modulates the Expression and Activation of Pattern Recognition Receptors in Mast Cells

**DOI:** 10.3390/ijms24129875

**Published:** 2023-06-08

**Authors:** Roberto Riquelme-Neira, Romina Walker-Vergara, Joan Antoni Fernández-Blanco, Patrocinio Vergara

**Affiliations:** 1 Núcleo de Investigaciones Aplicadas en Ciencias Veterinarias y Agronómicas, Facultad de Medicina Veterinaria y Agronomía, Universidad de Las Américas, Sede Concepción, Chacabuco 539, Concepción 4070254, Chile; rriquelmen@udla.cl (R.R.-N.); romina.walker@edu.udla.cl (R.W.-V.); 2 Department of Cell Biology, Physiology and Immunology, Universitat Autònoma de Barcelona, 08193 Barcelona, Spain; joanantoni.fernandez@gmail.com

**Keywords:** interleukin-10, MMC, PCMC, PRR, NOD2, TLR

## Abstract

Mast cells (MCs) are involved in several immune-related responses, including those in bacterial infections, autoimmune diseases, inflammatory bowel diseases, and cancer, among others. MCs identify microorganisms by pattern recognition receptors (PRRs), activating a secretory response. Interleukin (IL)-10 has been described as an important modulator of MC responses; however, its role in PRR-mediated activation of MC is not fully understood. We analyzed the activation of TLR2, TLR4, TLR7 and Nucleotide-binding oligomerization domain-containing protein 2 (NOD2) in mucosal-like MCs (MLMCs) and peritoneum-derived cultured MCs (PCMCs) from IL-10^−/−^ and wild-type (WT) mice. IL-10^−/−^ mice showed a reduced expression of TLR4 and NOD2 at week 6 and TLR7 at week 20 in MLMC. In MLMC and PCMC, TLR2 activation induced a reduced secretion of IL-6 and TNFα in IL-10^−/−^ MCs. TLR4- and TLR7-mediated secretion of IL-6 and TNFα was not detected in PCMCs. Finally, no cytokine release was induced by NOD2 ligand, and responses to TLR2 and TLR4 were lower in MCs at 20 weeks. These findings indicate that PRR activation in MCs depends on the phenotype, ligand, age, and IL-10.

## 1. Introduction

Mast cells (MCs) are long-lived, tissue-localized immune cells that originate from pluripotent progenitors in the bone marrow [1,2,3]. Immature mast cells migrate to peripheral tissues to complete their maturation and differentiation, mainly in those tissues that are in proximity to host-environment interfaces, such as the skin, gastrointestinal tract, and airways, having different functions and phenotypes depending on their location [4,5,6]. Accordingly, they are classified as mucosal MCs (MMC), which are found predominantly in the mucosa of the airways and the gastrointestinal tract, and connective tissue MCs (CTMC), mainly located in the skin and peritoneal cavity. In these locations, MCs act as sentinel cells, initiating immune responses to exogenous stimuli [6,7,8,9,10].

Mast cells play a crucial role in the maintenance of many physiological functions, such as vasodilation, homeostasis, tissue remodeling, angiogenesis, activation, mediator release, and the development and amplification of the inflammatory response and antimicrobial defense. However, the mechanisms of immunological interactions are still not fully understood [11,12,13,14,15,16,17]. At the same time, MCs are involved in the pathophysiology of multiple diseases including allergies, asthma, cardiovascular diseases, pulmonary fibrosis, osteoporosis, autoimmune arthritis, multiple sclerosis, mast cell activation syndromes, neoplastic diseases, inflammatory bowel syndrome, and inflammatory bowel disease (IBD) [14,18,19,20,21,22,23,24,25,26,27].

Moreover, in the particular case of IBD, it has been suggested that IL-10 modulates the interaction of the host with the microbiota [28,29,30]. In this sense, mice deficient in interleukin 10 (IL-10^−/−^) spontaneously develop enterocolitis under conventional housing conditions after 12 weeks of age, which is then used as a model of IBD [31,32,33]. This could be explained by the fact that IL-10 would be a major immunosuppressive and anti-inflammatory factor that is indispensable for the homeostatic control of infection and inflammation [30,34].

Previous studies on the relationship between MCs and IL-10 have shown that IL-10 regulates the proliferation and production of cytokines and the differentiation of MCs [35,36,37]. In addition, mice that are deficient in both MCs and IL-10 exhibit exacerbated colitis compared to single IL-10 knockout mice, which clearly reinforces the relationship between MCs and IL-10 [26,38]. Considering that MCs are highly heterogeneous according to their origin and location in the body, more information is needed regarding the role of IL-10 in different types of MCs.

Simultaneously, as part of their innate immunity, MCs play a pivotal role in inflammation and antimicrobial immunity mediated by pattern recognition receptors (PRRs) and can thus identify both pathogen-associated molecular patterns (PAMPs) and damage-associated molecular patterns (DAMPs) [39,40,41]. Via PRRs, MCs can recognize and respond to bacteria and viruses located both extracellularly and intracellularly [24,42,43,44,45,46,47]. Once activated after PAMP-PRR interactions, MCs release a variety of biologically active mediators, such as proteases, cytokines, and chemokines [25,39,44,48,49,50,51]. These mediators induce a diversity of biological effects, including the recruitment and activation of inflammatory cells, smooth muscle contractions, and an increase in vascular permeability [8,26,52]. In addition, responses to PRR activation are conditioned by the level of expression of PRRs in different MC subtypes [44]. Therefore, it is necessary to analyze each specific MC subpopulation individually to properly understand their modulation by PRRs.

The aim of this study was to characterize the effect of IL-10 deficiency on the MC phenotype and its activation via PRRs in stages before and after the development of colitis in a murine model of IBD.

## 2. Results

### 2.1. Morphological Analysis and mMCP-1 and mMCP-6 Characterization of MLMC and PCMC

MLMCs and PCMCs displayed typical MC phenotypes. There was no obvious morphologic difference between WT and IL-10^−/−^ MCs (Figure S1).

**Figure 1:**
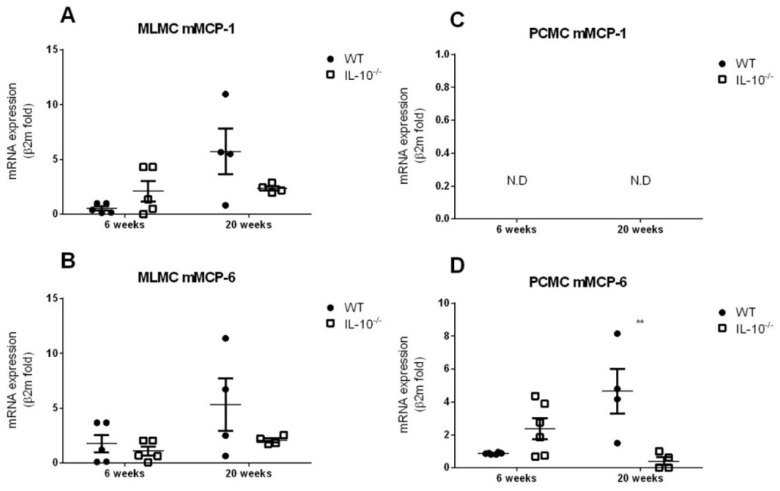
Gene expression of mMCP-1 and mMCP-6 by MLMCs and PCMCs derived from wild type and IL-10^−/−^ mice. Gene expression of mouse mast cell protease (mMCP)-1 (**A**,**C**) and mMCP-6 (**B**,**D**) was assessed in mucosal-like mast cells (MLMCs; **A**,**B**) and peritoneum-derived cultured mast cells (PCMCs; **C**,**D**). Relative expression of mMCPs mRNA was analyzed by quantitative reverse transcription-PCR and normalized to transcript levels of β-2-microglobulin. Expression levels were measured in wild type (WT; filled circles) and IL-10^−/−^ (empty squares). All the analyses were performed using cells isolated from 6 (left panels) and 20 (right panels) week old mice. Data are mean ± standard error of 4–6 animals per group. ** *p* < 0.01 vs. the wild type, 2-way ANOVA with Bonferroni posttest. N.D.: not detected.

The expression of mMCP-1 and mMCP-6 was similar in both genotypes from MLMC, with a higher expression (~1.5 folds) of each at 20 weeks in the WT animals. (Figure 1A,B). In PCMCs, no expression of mMCP-1 was detected, whereas expression of mMCP-6 was similar in both genotypes isolated from 6-week-old mice. However, in knockout PCMCs at 20 weeks, lower mMCP-6 expression was detected compared to age-matched WT (4.65 ± 1.369 vs. 0.405 ± 0.246 folds; *p* < 0.01) (Figure 1C,D).

### 2.2. Effects of IL-10 Deletion on TLR2 Expression and Release of Inflammatory Mediators Related to Its Activation by Pam_2_CSK4 in MLMC and PCMC

**Figure 2:**
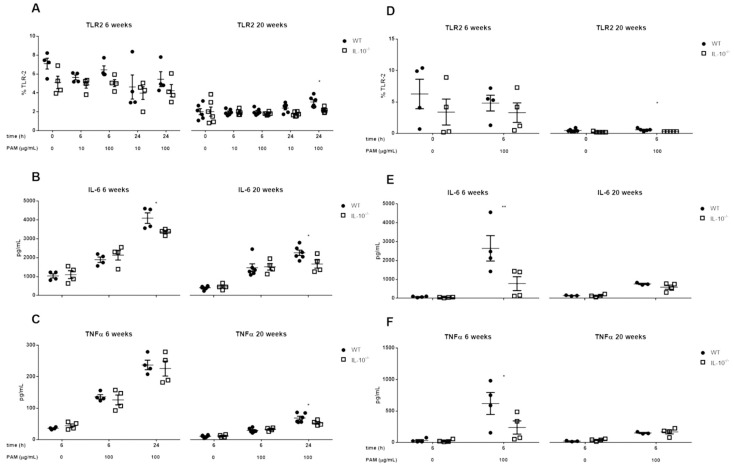
Effects of IL-10 deletion on TLR2 expression and release of inflammatory mediators related to its activation by Pam_2_CSK4 in MLMCs and PCMCs. The expression of toll-like receptor 2 (TLR2) was assesses in mucosal-like mast cells (MLMCs; **A**) and peritoneum-derived cultured mast cells (PCMCs; **D**). The percentage of cells positive for TLR2 was characterized in basal conditions (unstimulated; time 0) and after the stimulation with synthetic diacylated lipoprotein Pam_2_CSK4 (PAM). IL-6 (**B**,**E**) and TNFα (**C**,**F**) levels were measured in wild type (WT; filled circles) and IL-10^−/−^ (empty squares) MLMC (**B**,**C**) and PCMC (**E**,**F**) left unstimulated or stimulated with PAM. All the analyses were performed using cells isolated from 6 (**left panels**) and 20 (**right panels**) week old mice. Data are mean ± standard error of 4–6 animals per group. * *p* < 0.05, ** *p* < 0.01 vs. wild type, 2-way ANOVA with Bonferroni posttest.

No significant differences in TLR2 expression were observed in MLMCs and PCMCs under basal conditions (time zero, unstimulated) for both genotypes (WT and IL-10^−/−^ mice), but TLR2 expression was decreased in 20-week-old compared with 6-week-old mice (−70% WT; −60% IL-10^−/−^). When analyzing the effect of IL-10 deletion on TLR2 expression and inflammatory mediator release in MLMCs and PCMCs treated for 6 and 24 h with Pam_2_CSK4, respectively, a lower percentage of TLR2+ was observed after stimulation in IL-10^−/−^ cells compared with WT cells (*p* < 0.05) (Figure 2A,D).

Regarding inflammatory cytokine secretion (IL-6 and TNFα), deletion of IL-10 significantly reduced IL-6 secretion at 6 and 20 weeks and TNFα secretion at 20 weeks, in MLMC (Figure 2B,C; *p* < 0.05), and secretion of both cytokines was lower in knockout PCMC at week 6 (2644 ± 673 vs. 774 ± 367 pg/mL for IL-6; 619 ± 174 vs. 237 ± 105 pg/mL for TNFα) (Figure 2E,F). When comparing both populations of MCs, higher TNFα was observed by PCMCs compared to MLMC (619 ± 174 vs. 136 ± 7 pg/mL for WT; 237 ± 105 vs. 126 ± 15 pg/mL for IL-10^−/−^ at 6 h). Of note, when MLMC and PCMC were subjected to any of the stimuli used in the study, it was not possible to detect at the protein level IL-10, IL-1β, IL-12p70, or IFN-γ.

### 2.3. Effects of IL-10 Deletion on TLR4 Expression and Release of Inflammatory Mediators Related to Its Activation by LPS in MLMC and PCMC

**Figure 3:**
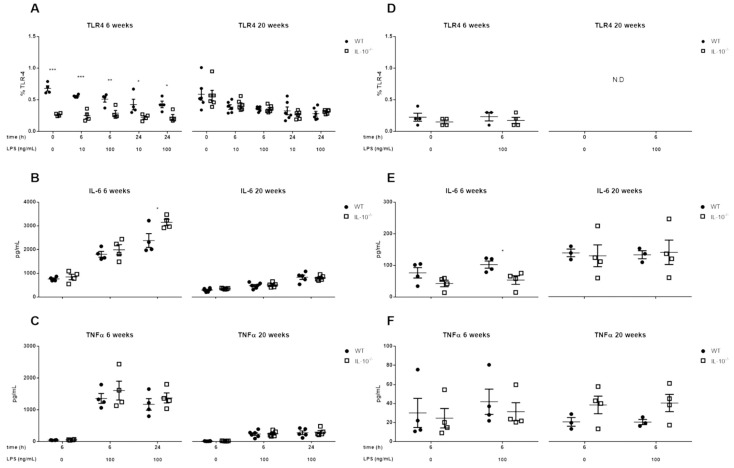
Effects of IL-10 deletion on TLR4 expression and release of inflammatory mediators related to its activation by LPS in MLMCs and PCMCs. The expression of toll-like receptor 4 (TLR4) was assesses in mucosal-like mast cells (MLMCs; **A**) and peritoneum-derived cultured mast cells (PCMCs; **D**). The percentage of cells positive for TLR4 was characterized in basal conditions (unstimulated; time 0) and after the stimulation with lipopolysaccharide (LPS) from *Escherichia coli* O55:B5. IL-6 (**B**,**E**) and TNFα (**C**,**F**) levels were measured in wild type (WT; filled circles) and IL-10^−/−^ (empty squares) MLMCs (**B**,**C**) and PCMCs (**E**,**F**) left unstimulated or stimulated with LPS. All the analyses were performed using cells isolated from 6 (**left panels**) and 20 (**right panels**) week old mice. Data are mean ± standard error of 4–6 animals per group. * *p* < 0.05, ** *p* < 0.01 and *** *p* < 0.001 vs. wild type, 2-way ANOVA with Bonferroni posttest. N.D.: not detected.

The basal proportion of MLMC expressing TLR4 on their surface was lower in knockout mice at 6 weeks (*p* < 0.001), but this difference was no longer apparent at week 20, becoming similar in both genotypes (~0.6%). In the unstimulated PCMC, no differences were observed at week 6, with TLR4 expression being undetectable at week 20 (Figure 3A,D). After TLR4 stimulation with LPS in MLMCs, the differences between both genotypes were maintained (*p* < 0.05). As for MLMCs, LPS did not affect TLR4 surface expression in PCMC.

Regarding the secretion of proinflammatory mediators, TLR4 activation induced the release of IL-6 and TNFα by MLMC but not by PCMC (Figure 3). When both genotypes were compared, MLMC derived from 6-week-old IL-10^−/−^ mice secreted more IL-6 after TLR4 stimulation than WT animals of the same age (Figure 3B; *p* < 0.05). On the other hand, at week 20, no differences in IL-6 secretion were observed. TNFα secretion by MLMC was not influenced by IL-10 and, although it did not reach statistical significance, it tended to be higher at week 20 in the case of IL-10^−/−^ PCMC (Figure 3C,F). Furthermore, a reduction in the proinflammatory response of MLMC to LPS was observed at 20 weeks compared to 6 weeks (approximately more than 1000 pg/mL for both cytokines).

### 2.4. Effects of IL-10 Deletion on TLR7 Expression and Release of Inflammatory Mediators Related to Its Activation by Imiquimod in MLMC and PCMC

**Figure 4:**
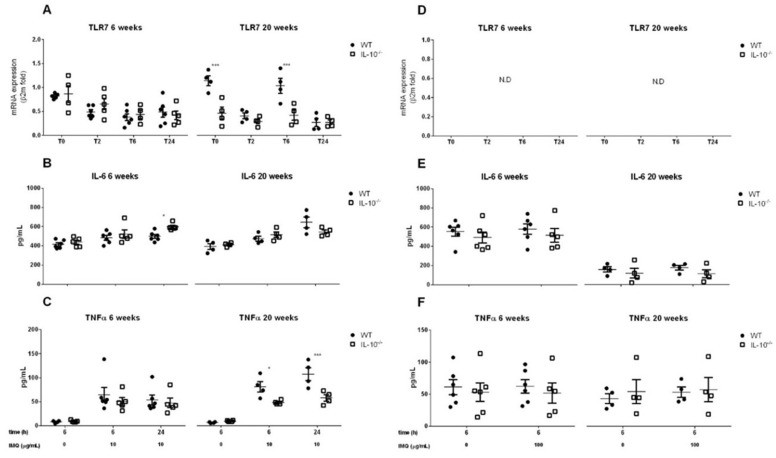
Effects of IL-10 deletion on TLR7 expression and release of inflammatory mediators related to its activation by Imiquimod in MLMCs and PCMCs. Gene expression of toll-like receptor 7 (TLR7) was assesses in mucosal-like mast cells (MLMCs; **A**) and peritoneum-derived cultured mast cells (PCMCs; **D**). Relative expression of TLR7 mRNA was analyzed by quantitative reverse transcription-PCR and normalized to transcript levels of β-2-microglobulin in basal conditions (unstimulated; time 0) and after the stimulation with imiquimod (IMQ). IL-6 (**B**,**E**) and TNFα (**C**,**F**) levels were measured in wild type (WT; filled circles) and IL-10^−/−^ (empty squares) MLMCs (**B**,**C**) and PCMCs (**E**,**F**) left unstimulated or stimulated with IMQ. All the analyses were performed using cells isolated from 6 (**left panels**) and 20 (**right panels**) week old mice. Data are mean ± standard error of 4–6 animals per group. * *p* < 0.05 and *** *p* < 0.001 vs. wild type, 2-way ANOVA with Bonferroni post-test. N.D.: not detected.

In basal conditions, MLMC derived from 6-week-old mice of both genotypes showed similar levels of TLR7 expression. On the other hand, at week 20, the expression of TLR7 was lower in MLMC when IL-10 was not present (*p* < 0.001). In contrast to MLMC, TLR7 was not detected in PCMC isolated from any of the genotypes at any time point. (Figure 4A,D). Stimulation of MLMC with imiquimod was associated with a time-lapse of TLR7 gene expression in both genotypes at week 6 (*p* < 0.001), and lower TLR7 expression in IL-10^−/−^ cells at 20 weeks (*p* < 0.001), like that observed in basal conditions.

The proinflammatory pattern depended on the genotype of the cells and the age of the animals from which they were isolated. At week 6 and after 24 h of stimulation, IL-6 levels were higher in MLMC obtained from IL-10^−/−^ mice (597 ± 15 vs. 498 ± 20 pg/mL). In contrast, at week 20, TNFα secretion in response to imiquimod was higher in WT MLMC (107 ± 13 vs. 57 ± 6 pg/mL at 24 h) (Figure 4B,C). Finally, consistent with the lack of detection of TLR7 in PCMC, we did not observe any effect of imiquimod on proinflammatory cytokine secretion (Figure 4E,F).

### 2.5. Effects of IL-10 Deletion on NOD2 Expression and Release of Inflammatory Mediators Related to Its Activation by MDP in MLMC and PCMC

**Figure 5:**
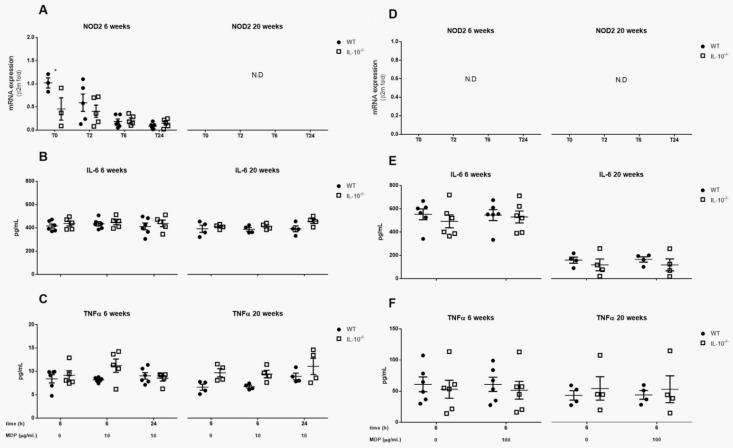
Effects of IL-10 deletion on NOD2 expression and release of inflammatory mediators related to its activation by MDP in MLMCs and PCMCs. Gene expression of nucleotide-binding oligomerization domain-containing protein 2 (NOD2) was assesses in mucosal-like mast cells (MLMCs; **A**) and peritoneum-derived cultured mast cells (PCMCs; **D**). Relative expression of NOD2 mRNA was analyzed by quantitative reverse transcription-PCR and normalized to transcript levels of β-2-microglobulin in basal conditions (unstimulated; time 0) and after the stimulation with muramyldipeptide (MDP). IL-6 (**B**,**E**) and TNFα (**C**,**F**) levels were measured in wild type (WT; filled circles) and IL-10^−/−^ (empty squares) MLMCs (**B**,**C**) and PCMCs (**E**,**F**) left unstimulated or stimulated with MDP. All the analyses were performed using cells isolated from 6 (**left panels**) and 20 (**right panels**) week old mice. Data are mean ± standard error of 4–6 animals per group. * *p* < 0.05 vs. the wild type, 2-way ANOVA with Bonferroni posttest. N.D.: not detected.

The gene expression of the NOD2 receptor was only detected in MLMC isolated from 6-week-old mice. In this case, we observed a downregulation of gene expression in IL-10^−/−^ animals compared with WT mice (*p* < 0.05). However, these differences were no longer detected after cell culture and stimulation with MDP, which induced a reduction in NOD2 expression (Figure 5A,D).

In relation to cytokine release, no changes were observed in IL-6 or TNFα levels associated with the stimulation with MDP or with the expression of IL-10 in any MC subtype (Figure 5B,C,E,F).

### 2.6. Effects of the Lack IL-10 on FcεRI-Mediated Responses in MLMC and PCMC

**Figure 6:**
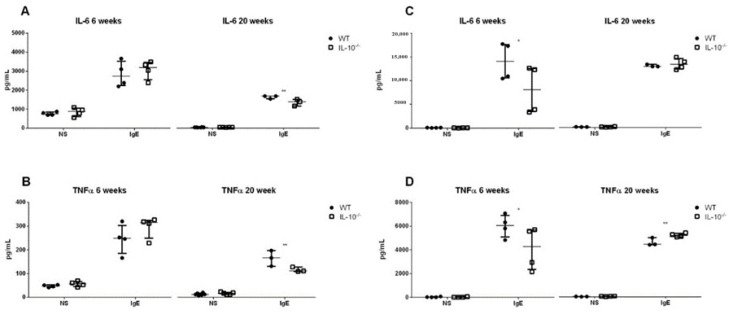
Effects of the lack IL-10 on FcεRI-mediated release of proinflammatory mediators in MLMCs and PCMCs. IL-6 (**A**,**C**) and TNFα (**B**,**D**) levels were measured in wild type (WT; filled circles) and IL-10^−/−^ (empty squares) MLMCs (**A**,**B**) and PCMCs (**C**,**D**) left unstimulated (NS) or stimulated with dinitrophenyl-human serum albumin. All the analyses were performed using cells isolated from 6 (**left panels**) and 20 (**right panels**) week old mice. Data are mean ± standard error of the mean of 3–4 animals per group. * *p* < 0.05 and ** *p* < 0.01 vs. the wild type, 2-way ANOVA with Bonferroni posttest.

No differences between genotypes were observed in MLMC at 6 weeks; however, FcεRI-mediated IL-6 and TNFα secretion was lower in IL-10^−/−^ cells at 20 weeks (*p* < 0.01) (Figure 6A,B). In PCMC, IL-10 deletion reduced cytokines levels detected after FcεRI activation at week 6 (*p* < 0.05). In contrast, when obtained at week 20, IL-10^−/−^ MCs produced similar levels of IL-6 and higher levels of TNFα compared to WT cells (5207 ± 83 vs. 4211 ± 195 pg/mL) (Figure 6B,D).

## 3. Discussion

IL-10 has been suggested to modulate mast cell functions and inflammatory responses related to host-microorganism interactions [26,31,53,54]. In the present study, we describe for the first time, the role of IL-10 in pattern recognition receptor expression and activation of MLMCs and PCMCs. Interestingly, both PRR expression and activation were found to be dependent on the MC subtype, the age of the animals, and conditioned by IL-10 deficiency.

As described in previous studies, both MLMC and PCMC stained positively for FcεRI and c-kit (CD117) [55,56]. In addition, mMCP-1, a protease typically associated with mucosal MCs, was detected in MLMCs but not in PCMCs [57,58]. This demonstrates that the protocol used to derive MLMC from bone marrow cells induces a differentiation to a mucosal MC phenotype [59]. It also confirms that mMCP-1 is a useful marker for distinguishing mucosal from connective tissue MCs in mice. On the other hand, mMCP-6, usually considered a marker for connective tissue MCs [20], was detected in both cultures. This could be explained by the expression of proteases generally associated with CTMC, including mMCP-6, by mucosal and transitional populations of MCs [20,60]. Regarding the influence of IL-10 in MC differentiation, while no obvious differences in morphology were detected between both genotypes, we show that IL-10 modulates mMCPs expression. As IL-10 can induce the expression of mMCPs, the lower expression of mMCP-6 detected in knockout MCs could be related to the absence of IL-10 and the lack of its autocrine activity [60,61].

According to the results obtained, the expression of PRRs could be related to the phenotype, origin, and differentiation of MCs, as has been described by other authors [44,62,63,64]. It was found that TLR2, TLR4, TLR7 and NOD2 were expressed by MLMC. However, in PCMC, only TLR2 and TLR4 (assessed at protein level by flow cytometry) could be detected. Although the percentage of MCs expressing TLR2 and TLR4 was low, it was sufficient to trigger a functional response after their stimulation with specific agonists. This supports the constitutive expression of TLR2 and TLR4 in MMC and CTMC, and the heterogeneity in TLR7 and NOD2 expression in different MC populations [44,51,62,63,64]. However, the exact proportion of MCs of each subtype expressing PRRs and the factors behind the modulation of their expression are more difficult to specify. It is important to note, that MLMC could show some degree of heterogeneity, as they are cells differentiated from bone marrow progenitors and could present transitional characteristics. This does not apply in the case of PCMC, as these are obtained as differentiated cells from the peritoneum. For instance, in our study, the proportion of MLMCs and PCMCs expressing TLR2 was similar. In contrast, Mrabet-Dahbi et al. (2009) [44] found that the percentage of TLR2^+^ cells was higher in PCMCs than in bone marrow cultured MCs, used as a model of MMCs with a higher percentage of PCMCs expressing TLR2 and TLR4. The difference detected between their results and ours could be related to changes in the in vivo model and the in vitro maturation of the cells. For instance, they cultured PCMC for 9 days in comparison to our 28-day culture protocol. In addition, we show for the first time how PRR expression evolves with aging in MCs, similar to what others have described for macrophages [65]. We observed that the proportion of TLR2^+^ and TLR4^+^ PCMC was higher when derived from younger mice. Consequently, the results obtained by Mrabet-Dahbi et al. [44] could be conditioned by the age of the animals used to isolate cells. Unfortunately, this information was not specified in their manuscript. That said, it seems clear that both in vivo and in vitro variables modulate the expression of PRRs in MCs and this should be considered in future experiments.

When we characterized the role of IL-10 in PRR expression, we observed that it depended on the receptor and the age of the animals when cells were isolated. In the case of MLMCs, IL-10 deletion reduced the expression of TLR4 and NOD2 at week 6 and TLR7 at week 20. Regarding CTMCs, IL-10 could have contributed to the observed TLR2 expression by week 20, a time point when the other PRRs evaluated were not detected in this cell type. To date, the mechanisms by which IL-10 regulates PRRs have not been characterized. However, the modulation by IL-10 of the repertoire of PRRs expressed by MCs could be involved in the ability of this cytokine to induce MC expansion, survival, and activation [66,67]. Therefore, it would be of interest to evaluate the possible regulation of IL-10 in the signaling pathways associated with the activation of PRRs, as described in previous studies, where the inhibitory effect of IL-10 on the production of inflammatory mediators has been mentioned [29].

After studying PRR expression in MCs, we characterized their activation and the consequent release of immune mediators. Firstly, Pam_2_CSK4-mediated activation of TLR2 induced the secretion of IL-6 and TNFα in both genotypes and MC subtypes. These results complement other studies in which a TLR2-dependent production of cytokines was observed in bone marrow-derived MCs and PCMC after their stimulation with the TLR2 agonists peptidoglycan and lipoteichoic acid [39,44,68]. However, we cannot exclude Pam_2_CSK4 recognition by a heterodimeric interaction of TLR2 with other TLRs, such as TLR1 or TLR6 [39,69]. Therefore, although out of the scope of the present study, the interaction among different TLRs in MCs should be addressed in future experiments.

TLR4 and TLR7 activation, as discussed above for TLR2, also induced the secretion of pro-inflammatory cytokines in WT and IL-10^−/−^ MLMC. In contrast, TLR4 and TLR7-mediated secretion of IL-6 and TNFα was not observed in PCMC. Although under LPS stimulation a slight production of IL-6 was detected in cells obtained at 20 weeks of age, this could be secreted constitutively, as no differences were observed between unstimulated and stimulated cells. While the lack of response of PCMC to the TLR7 ligand Imiquimod matches previous reports, the finding that TLR4 activation did not induce cytokine secretion was unexpected [44,62]. This could be related to a lower expression of TLR4 or the absence of membrane CD14 in the PCMC we used for our studies in comparison with those used by groups that observed a positive response [44,68].

Another relevant finding is the importance of age in the release of cytokines by MCs in response to TLR2 and TLR4 ligands. Thus, the reduction in IL-6 and TNF secretion observed at 20 weeks compared with 6-week-old mice could be attributed to the differences in TLR expression discussed above. At the same time, this could be associated with a general reduction in the production and secretion of proinflammatory mediators by MCs with age. This hypothesis is supported, at least in the case of MLMC, by the results obtained with IgE stimulation. As PRR activation and their regulation of immune responses changes with age, immune response regulated by PRRs would be affected. This would condition the interaction of the host with its microbiota. As a result, the secretion of cytokines would be reduced, facilitating the overgrowth of bacteria and their interaction with the epithelium. These mechanisms could be involved in the age-dependent activation and perpetuation of colitis observed in IL-10^−/−^ mice [26,31,65].

Finally, NOD2 ligands, according to our results and those obtained by others, do not induce the release of cytokines by either WT or IL-10^−/−^ MCs [51,64]. In general, this would be justified by the lack of expression of the receptor. In this respect, the expression of NOD2 depends on IFNγ, which we did not detect on our cell cultures [51]. On the other hand, this lack of expression could also be attributed to other factors such as differences in translation efficiency, mRNA stability, or defects in signal transduction pathways. Therefore, further assays to study NOD2 in MCs exposed to IFNγ would help to better understand PRR-mediated responses in inflammatory diseases.

Regarding the modulation of cytokine secretion by IL-10, it is noteworthy that the results obtained were different depending on the PRR evaluated, reducing, in the case of TLR2, the secretion of IL-6 and TNFα by MCs in the absence of IL-10. On the contrary, TLR4 ligands induced enhanced immune responses in cells derived from IL-10^−/−^ mice. This could be due to the potential role of IL-10 in modulating the expression of inflammatory mediators, as has been demonstrated for other cell types, including macrophages and monocytes [25,70,71,72,73]. Furthermore, it has even been described that in the context of LPS stimulation, IL-10 can produce an inhibition of NF-κB target genes in macrophages or interferon regulatory factor (IRF) in dendritic cells, probably triggering different signaling and response pathways [43,74]. Therefore, a complex and poorly understood scenario exists in which immune responses are a result of the counter-regulation of different PRRs by IL-10. Indeed, paradoxically, signal transducer and activator of transcription 3 (STAT3) activation can be triggered by both IL-10 and IL-6 despite resulting in a distinct gene activation program. In addition, we cannot rule out the possibility that the epigenetic landscape of genes regulated by pathways such as STAT3 may change in response to different stimuli, allowing the selective transcription of pro-inflammatory or anti-inflammatory mediators [43,75]. At the same time, the pro-inflammatory or anti-inflammatory roles of MCs are conditioned not only by the kind of stimulus and the MC subtype but also by their tissue location and interactions with other components of the immune system [54,76].

In summary, these results show that both mucosal and connective tissue MCs have the potential to respond to a variety of microorganisms via the activation of a diverse repertoire of PRRs, leading to the secretion of different cytokines depending on the stimulated PRRs, the MC subtype, and the age of the individuals from which MCs or their precursors are isolated.

Finally, the present study provides new insights into the role of IL-10 in the expression of PRRs and cytokine production in differentiated MCs to aid in the understanding of inflammatory processes in which the microorganism-host interaction seems to be key, such as IBD.

## 4. Materials and Methods

### 4.1. Animals

B6.129P2-*Il10^tm1Cgn^*/J (IL-10^−/−^) and C57BL/6J WT female mice were obtained from The Jackson Laboratory (Bar Harbor, ME, USA) (5-week-old, weight 15–17 g). Upon arrival, animals were randomly divided into four groups [6-week-old WT (*n* = 4), 6-week-old IL-10^−/−^ (*n* = 4), 20-week-old WT (*n* = 6) and 20-week-old IL-10^−/−^ (*n* = 5)]. Mice were housed in individually ventilated cages under conventional conditions in an environmentally controlled room. Mice had access to tap water and a Teklad Global 14% protein rodent maintenance diet (ENVIGO CRS S.A., Oxfordshire, UK) ad libitum. All the experimental procedures were approved by the Ethics Committee of the Universitat Autònoma de Barcelona and the Generalitat de Catalunya (procedures 2773 and 8814, respectively).

### 4.2. Collection and Culture of Peritoneum-Derived Cultured Mast Cells (PCMC) and Mucosal-like Mast Cells (MLMC)

At the time of the experiments, mice were deeply anesthetized with isoflurane (Isoflo^®^, Madrid, Spain) and euthanatized by exsanguination through intracardiac puncture, followed by cervical dislocation. Immediately after, a peritoneal lavage was performed with 10 mL of sterile PBS to collect peritoneal mast cells, which were used to obtain peritoneum-derived cultured mast cells (PCMC), as previously described [53]. In addition, intact femurs and tibias were removed from the mice, and the bone marrow was washed using a 23-gauge needle and a 5 mL syringe filled with Dulbecco’s Modified Eagle Medium (DMEM), supplemented with 10% fetal calf serum (FCS), 1% penicillin/streptomycin, 1 mM sodium pyruvate, and 2 mM L-glutamine (DMEM/FCS) (Thermo Fisher Scientific©, Waltham, MA, USA). Then, mucosal-like mast cells (MLMC) were cultured and differentiated as previously described [54].

After 9 days and 4 weeks, MLMC and PCMC were harvested with at least 85% and 99% of purity, respectively, and their viability was monitored using Trypan blue staining, and their surface expression of c-kit and FcεRI was measured by flow cytometry.

### 4.3. Cytospin and Staining of Mast Cell

Cellular suspensions obtained from MLMC and PCMC cultures were cytocentrifuged using a Cytocentrifuge Cytospin^TM^ 4 (Thermo Fisher Scientific©, Waltham, MA, USA) onto a slide (600 rpm, 6 min, room temperature), air-dried for 15 min, fixed in Carnoy’s fixative solution for 15 min, and stained with a 1% Toluidine Blue O solution (Sigma-Aldrich^®^, St. Louis, MO, USA) at pH = 7 for 5 min. Samples were dehydrated and mounted in DPX medium.

### 4.4. In Vitro Cell Stimulation

MLMCs and PCMCs were seeded in 12-well flat-bottom plates (Nunclon^®^, Thermo Fisher Scientific©, Waltham, MA, USA) in triplicate at a density of 5 × 10^5^ cells/mL in complete culture medium in a humidified 10% CO_2_ incubator at 37 °C. To study responses related to FcεRI activation, cells were sensitized with 1 µg/mL anti-dinitrophenyl IgE (clone: SPE-7; ref: D8406, Sigma-Aldrich^®^, St. Louis, MO, USA) overnight, and then challenged for 6 h with 50 ng/mL dinitrophenyl-human serum albumin (DNP-HSA; Sigma-Aldrich^®^, St. Louis, MO, USA). To evaluate TLR2, TLR4, TLR7, and NOD2-mediated responses, cells were stimulated with Pam_2_CSK4 (InvivoGen™, San Diego, CA, USA; 10 and 100 µg/mL), lipopolysaccharide (LPS) from *Escherichia coli* serotype O55:B5 (Sigma-Aldrich^®,^ St. Louis, MO, USA; 10 and 100 µg/mL), imiquimod (InvivoGen™, San Diego, CA, USA; 10 and 100 µg/mL), and muramyl dipeptide (MDP; InvivoGen™, San Diego, CA, USA; 10 and 100 µg/mL), respectively. TLR2 and TLR4 were assessed at the protein level by flow cytometry; TLR7 and NOD2 were assessed at the mRNA level by RT-qPCR. MLMC were stimulated for 6 and 24 h, PCMC were stimulated for 6 h. Before and after stimulation, cells were collected by centrifugation (250× *g*, 7 min, room temperature) and used to perform morphology and flow cytometry analyses. Cells and supernatants were frozen and stored at −80 °C until analysis.

### 4.5. RNA Extraction and Reverse Transcription Quantitative Polymerase Chain Reaction (RT-qPCR)

Total RNA from MLMC and PCMC was extracted using the RNeasy^®^ Mini Kit (Qiagen, Germany). RNA was quantified by Nanodrop (NanoDrop™ Technologies, Wilmington, DE, USA) and 1 µg of RNA was reverse-transcribed in a 20 µL reaction volume for cDNA synthesis using the iScript^TM^ cDNA Synthesis kit (Bio-Rad Laboratories Inc., Hercules, CA, USA). The temperature profile for reverse transcription was 25 °C for 5 min, 42 °C for 30 min, and 85 °C for 5 min.

Validated TaqMan^®^ gene expression assays with hydrolysis probes for mouse mast cell proteases (mMCPs), cytokines, PRRs, and reference genes were used (Applied Biosystems™, Waltham, MA, USA; Table S1). PCR reaction mixtures were transferred to clear 384-well reaction plates (Bio-Rad Laboratories Inc., Hercules, CA, USA); sealed by adhesives and C1000 Touch™ Thermal Cycler platform (Bio-Rad Laboratories Inc.) for 40 cycles (95 °C for 15 s, 60 °C for 1 min). Fluorescence signals measured during amplification were processed after amplification. Bio-Rad CFX Manager 2.1 software was used to obtain the cycle threshold (CT) for each sample. Each sample was run in triplicate and data were analyzed by the comparative CT method [2^−∆∆CT^], as previously described [77]. Actin Beta (ACTB) and β-2-microglobulin were tested as reference genes. β-2-microglobulin expression levels were used for normalizing the mRNA levels of the target genes because of their constancy across the different experimental groups. Controls of analytical specificity included omission of reverse transcriptase to exclude contamination with genomic DNA, and no-template controls, omitting the cDNA.

### 4.6. Flow Cytometry

Next, 5 × 10^5^ PCMCs and MLMCs were harvested, washed twice in flow cytometry staining buffer (FACS buffer), and pre-incubated for 15 min with 200 µL of FACS buffer with 1 µg of purified anti-mouse CD16/CD32 (clone: 93; ref: 14-0161) to block non-specific binding to Fc Receptor (FcR). Without washing, 50 µL (1.25 × 10^5^) of pre-incubated cells were simultaneously stained for cell surface markers for 30 min at 4 °C by adding 50 µL of a mix of monoclonal antibodies (eBioscience Inc., San Diego, CA, USA), anti-mouse CD117 (c-Kit) conjugated with allophycocyanin (clone: 2B8; ref: 17-1171), anti-mouse Fc epsilon Receptor I alpha (FcεR1α) conjugated with fluorescein isothiocyanate (clone: MAR-1; ref: 11-5898), and anti-mouse CD282 (TLR2) (clone: 6C2; ref: 12-9021) or anti-mouse CD284 (TLR4) conjugated with phycoerythrin (clone: UT41; ref: 12-9041). For TLR2 and TLR4, Rat IgG2b (clone: A95-1; ref: 12-4032), and Mouse IgG1 (clone: P3.6.2.8.1; ref: 12-4714) were used as isotype controls, respectively. All the antibodies were previously titrated. After two washes, cells were resuspended in 0.2 mL of FACS buffer and the analysis of 20,000 events was performed using a six-color BD FACSCanto flow cytometer and BD FACSDiva Software v7.0 (BD Biosciences™, Franklin Lakes, NJ, USA). The strategy of gating used was as follows: Firstly, a gate of live cells based on forward and side scatter properties was set, followed by a second gate on the CD117 and FcεR1 double positive population to select MCs. Unstained cells and fluorescence minus one control (only CD117 and FcεR1 antibodies) were used to set the negative controls for TLRs on MCs.

### 4.7. Quantification of Cytokines Concentration

The concentration of cytokines IL-1β, IL-6, IL-10, IL-12p70, IFN-γ, MCP-1, and TNFα in cultured PCMC and MLMC supernatants was measured using a cytometric bead analysis Mouse Inflammation Kit (BD Biosciences™, Franklin Lakes, NJ, USA). Data were obtained using FACScan and analyzed with the BD™ Cytometric Bead Array (CBA) Analysis Software v3.0 (BD Biosciences™, Franklin Lakes, NJ, USA).

### 4.8. Data Analysis

Data were expressed as mean ± standard error of the mean (SEM). Statistical analysis was performed using GraphPad Prism v.6.0 software (Graph Prism Software Inc., San Diego, CA, USA). Comparisons between multiple groups were performed using one-way ANOVA or two-way ANOVA, followed when necessary by a Bonferroni multiple-comparison test. *p*-values < 0.05 were considered statistically significant.

## Data Availability

The datasets generated during and/or analyzed during the current study are available from the corresponding author on reasonable request.

## Supplementary Materials

The following supporting information can be downloaded at: https://www.mdpi.com/article/10.3390/ijms24129875/s1.
