# Supplementary material for: IL-10 Modulates the Expression and Activation of Pattern Recognition Receptors in Mast Cells
Source: Int J Mol Sci. 2023 Jun 8;24(12):9875. doi: 10.3390/ijms24129875 (PMC10298310; doi:10.3390/ijms24129875)
Supplement: Supplementary file 1 [file ijms-24-09875-s001.zip › ijms-2392298-supplemenatry materials.pdf]

Supplementary Materials

Table S1. TaqMan® gene expression assays.

Protein	Gene symbol	Assay reference
Beta-2-microglobulin	*B2m*	Mm00437762_m1
Beta-actin	*Actb*	Mm00607939_s1
Interleukin 1 beta (IL-1β)	*Il1b*	Mm00434228_m1
Interleukin 6 (IL-6)	*Il6*	Mm00446190_m1
Tumor necrosis factor-alfa (TNFα)	*Tnf*	Mm00443258_m1
Mast cell protease 1 (mMCP-1)	*Mcpt1*	Mm00656886_g1
Mast cell protease 6 (mMCP-6)	*Tspb2*	Mm01301240_g1
Nucleotide-binding oligomerization domain-containing protein 2	*Nod2*	Mm00467543_m1
Toll-like receptor 7	*Tlr7*	Mm00446590_m1

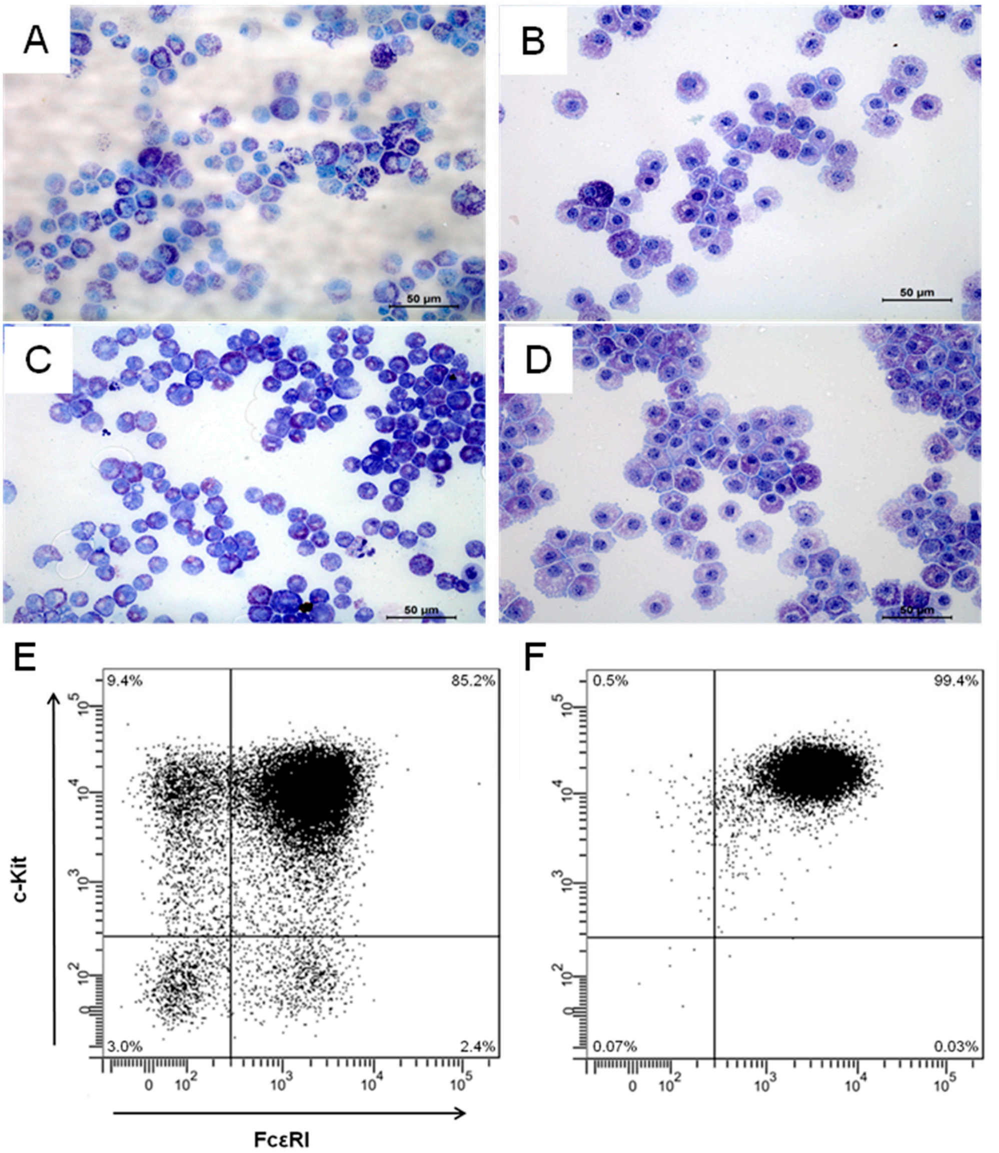

Figure S1. Characterization of the morphology and purity of MLMCs and PCMCs isolated from wild type and IL-10$^{-/-}$ mice. The images correspond to Toluidine blue-stained cytospin preparations of mucosal-like mast cells (MLMCs; A, C) and peritoneum-derived cultured mast cells (PCMCs; B, D) from wild type (A, B) or IL-10$^{-/-}$ (C, D) mice. Toluidine blue-positive cells contain dark blue cytoplasmic granules. Original magnification: 40X. Scale bars: 50 µm. (E, F) Two-color FACS® analysis of high-affinity IgE receptor (y-axis) and c-kit (x-axis) expression of MLMCs (E) or PCMCs (F) from wild type and IL-10$^{-/-}$ mice, respectively (similar results were obtained independently of the genotype).
